# Flexibility That Retains: How Scheduling Practices Influence Nurses’ Decisions to Stay

**DOI:** 10.1155/jonm/1548034

**Published:** 2026-08-31

**Authors:** Razan Odaibat, Ghada Abu Shosha, Islam Oweidat, Mutaz I. Othman, Hana A. Alsaeed, Khalid Al-Mugheed, Amany Anwar Saeed Alabdullah, Sally Mohammed Farghaly Abdelaliem

**Affiliations:** ^1^ Faculty of Nursing, Zarqa University, Zarqa, Jordan, jadara.edu.jo; ^2^ Maternal and Child Health Department, Faculty of Nursing, Zarqa University, Zarqa, Jordan, jadara.edu.jo; ^3^ Community and Mental Health Nursing Department, Faculty of Nursing, Zarqa University, Zarqa, Jordan, jadara.edu.jo; ^4^ Nursing Department, Hamad Medical Corporation, Doha, Qatar, hamad.qa; ^5^ Clinical Nursing Department, College of Nursing, University of Mosul, Mosul 41001, Nineveh Governorate, Iraq, uomosul.edu.iq; ^6^ College of Nursing, Riyadh Elm University, Riyadh, Saudi Arabia, riyadh.edu.sa; ^7^ Department of Maternity and Pediatric Nursing, College of Nursing, Princess Nourah bint Abdulrahman University, P.O. Box 84428, Riyadh 11671, Saudi Arabia, pnu.edu.sa; ^8^ Department of Nursing, Faculty of Allied Health Sciences, Kuwait University, Kuwait City, Kuwait, kuniv.edu

**Keywords:** hospitals and Jordan, nurse manager, retention, scheduling

## Abstract

**Background:**

Nurse retention is a pressing issue globally, particularly in Jordan, where governmental hospitals face significant challenges in maintaining a stable nursing workforce.

**Aim:**

This study aims to investigate the relationship between NMs’ scheduling flexibility and nurse retention.

**Methods:**

A cross‐sectional correlational study was conducted involving 155 nurses from five governmental hospitals across Jordan. Data were collected from 1 October–1 November, 2024 using the Work Schedule Flexibility Survey (WSFS) and the Revised Casey–Fink Nurse Retention Survey.

**Results:**

Nurses reported low levels of scheduling flexibility (*M* = 1.52, SD = 0.28) and retention factors (*M* = 49.9, SD = 10.5). Scheduling flexibility was positively correlated with job retention (*r* = 0.79, *p* < 0.01). Age and years of nursing experience were significant positive predictors of retention, whereas excessive night shifts, increased workload, and demanding work schedules were significant negative predictors.

**Conclusion:**

Improving scheduling flexibility, reducing workload, and strengthening managerial support may enhance nurse retention. This study provides context‐specific evidence from Jordanian nurses to inform workforce policies and sustainable retention strategies in health care.

**Implications for Nursing Management:**

Flexible scheduling is an important nursing management strategy that enhances nurses’ job satisfaction and well‐being. Since high turnover rates remain a global challenge, nurse managers should promote flexible work arrangements to improve work–life balance, reduce burnout, and strengthen staff retention. Such practices can also enhance organizational commitment and quality of patient care.

## 1. Introduction

The World Health Organization (WHO) projects a global shortage of 4.5 million nurses by 2030, highlighting the urgent need to improve nurse retention to sustain high‐quality healthcare services worldwide [1]. As the largest component of the healthcare workforce, nurses play a pivotal role in delivering safe patient care, improving clinical outcomes, and maintaining the stability of health systems [2]. However, nurse turnover remains a persistent global challenge, driven by excessive workload, inadequate compensation, limited professional development opportunities, and inflexible work schedules [3].

### 1.1. Background

Scheduling flexibility has emerged as an important organizational strategy for improving nurses’ job satisfaction, work–life balance, and retention. Flexible and responsive scheduling enable nurses to better balance professional and personal responsibilities, reduce work‐related stress and burnout, and enhance overall well‐being. The implementation of information technology‐based scheduling systems has further improved scheduling efficiency, reduced administrative burden for nurse managers, and increased staff satisfaction [4]. International evidence indicates that inflexible scheduling is associated with higher turnover intentions. For example, a large quantitative study involving more than 500 nurses found that rigid scheduling increased turnover intentions by approximately 30% [5]. Moreover, nurse turnover imposes substantial financial costs on healthcare organizations through recruitment, orientation, and overtime expenditures [6].

In Jordan, nurse retention has become a critical workforce issue. The country faces an ongoing nursing shortage together with a turnover rate estimated at 36.6%, placing considerable pressure on healthcare organizations and threatening the quality and continuity of patient care [7]. Previous Jordanian studies have identified excessive workload, poor scheduling practices, limited autonomy, and unfavorable working conditions as important contributors to nurses’ intention to leave the profession [8]. Despite these challenges, empirical evidence examining how scheduling flexibility influences nurse retention in Jordan remains limited. Regional evidence also supports the importance of organizational factors in retaining nurses. Studies conducted in Egypt have demonstrated that work–life balance and transformational leadership positively influence nurse retention [9]. Research from Lebanon identified shorter working hours, scheduling autonomy, and effective teamwork as significant predictors of nurses’ intention to remain in their positions [10]. Similarly, studies have shown that supportive leadership and flexible scheduling practices improve job satisfaction and reduce turnover intentions [11, 12]. Nevertheless, few studies have specifically investigated scheduling flexibility implemented by nurse managers within the Jordanian healthcare context. Given the persistent nursing shortage, high turnover rates, and limited evidence from Jordan, investigating the relationship between nurse managers’ scheduling flexibility and nurse retention is both timely and necessary. This study addresses an important knowledge gap by examining how scheduling flexibility relates to nurse retention among Jordanian nurses while accounting for workload and demographic characteristics. The findings were expected to provide context‐specific evidence to inform workforce policies and scheduling practices aimed at improving nurse retention and strengthening the sustainability of the Jordanian healthcare system.

## 2. Methods

### 2.1. Design of the Study and Data Collection

The study utilized a descriptive design with a correlational cross‐sectional approach to effectively assess the level of scheduling flexibility and its impact on nurse retention rates, with data collected at a single point in time to provide a snapshot of the variables involved. This study was conducted across five governmental institutions.

### 2.2. Participants and Sampling Procedure

The target population includes registered nurses, those with at least 6 months of experience, and those willing to participate. Exclusion criteria include temporary or per diem contracts, nonclinical roles, and those planning to retire within 6 months. The sampling procedure used convenience sampling, allowing for a large sample size without compromising the study’s practicability. The sample size was calculated using G∗Power3.1.9.7 software, a multiple regression analysis, with a medium effect size of 0.15 and seven predictors. The final sample size was 155 nurses.

### 2.3. Instruments

This study collected sociodemographic, work‐related, and organizational variables related to nurse scheduling flexibility and retention. Continuous variables included age, nursing experience, and working hours. Categorical variables included education level, gender, marital status, and department.

The Work Schedule Flexibility Survey (WSFS) is a reliable tool designed to measure nurses’ perceived level of work schedule flexibility. It consists of four questions, each addressing specific aspects of scheduling flexibility [13]. The responses to the four questions are summed, and the total score is normalized to yield an overall perceived flexibility score ranging from 0 to 4. Higher scores reflect greater perceived scheduling flexibility, with a score of 0 indicating no flexibility and a score of 4 indicating maximum flexibility. The WSFS is both reliable and valid, as evidenced by its high Cronbach’s alpha coefficient of 0.83, which demonstrates strong internal consistency [13].

The Revised Casey–Fink Nurse Retention Survey (RCFNRS) is a comprehensive tool designed to evaluate factors influencing nurse retention, including job satisfaction, professional support, work environment, and adaptability in work arrangements [14]. It comprises 34 items distributed across four subscales: Recognition/Rewards, Professional Nursing Role, Mentoring, and Work Adaptability. The survey uses a four‐point Likert scale to calculate scores for each subscale and a total retention score. The RCFNRS is both reliable and valid with Cronbach’s alpha of 0.84 and a CVI of 0.91 [14].

### 2.4. Ethics Approval

The study was approved by the Institutional Review Board (IRB) of the Faculty of Nursing, XXX University (Approval No. XXX) and by the IRB of the Ministry of Health (MOH), XXX. Written informed consent was obtained from all participants prior to data collection. All study procedures were conducted in accordance with the ethical principles of the Declaration of Helsinki and all applicable institutional guidelines and regulations.

### 2.5. Data Collection

Data collection was conducted between 1 October and 1 November, 2024. Nurse managers at each hospital assisted only with coordinating access to eligible nurses, identifying appropriate times for questionnaire distribution that minimized disruption to patient care, and facilitating communication with the research team. They were not involved in recruiting participants, obtaining consent, or accessing completed questionnaires to avoid potential coercion or response bias.

Eligible nurses were informed about the purpose of the study and those who agreed to participate provided written informed consent. Self‐administered paper questionnaires were distributed to participants during their work shifts in designated staff areas. Participants completed the questionnaires anonymously at their convenience and returned them in sealed envelopes to secure collection boxes placed in each hospital or directly to the research team. No personal identifiers were collected, and all completed questionnaires were stored securely to maintain confidentiality.

### 2.6. Statistical Analysis

The study followed rigorous statistical tests, including descriptive statistics, Pearson correlation analysis, and linear regression analysis, to ensure accuracy and completeness. Descriptive statistics provided a clear overview of the sample and variable distribution. Pearson correlation analysis tested linear relationships between variables, such as scheduling flexibility and nurse retention, identifying significant associations. Stepwise multiple linear regression was used to explore predictive relationships and measure the effect of predictors on nurse retention. Before conducting inferential analyses, normality of the continuous variables was tested by using visual inspection of histograms and Q–Q plots, as well as measuring the values of skewness and kurtosis. Linearity, homoscedasticity, and the independence of residuals were checked prior to performing the regression analysis. The presence of multicollinearity was checked using variance inflation factor (VIF) and tolerance. No violations of the assumptions were found. All statistical analyses were conducted using Statistical Package for the Social Sciences (SPSS) Statistics Version 26 software by IBM, with a significance level of p < 0.05, ensuring a thorough examination of the data and drawing meaningful conclusions.

## 3. Results

### 3.1. Demographic Characteristics

A total of 155 nurses participated in the study (response rate = 91%). The mean age of the participants was 32.5 years (SD = 5.1), and the mean years of nursing experience was 10.2 (SD = 3.4). About three‐quarters (74.2%) of the participants had bachelor’s degrees, 61.3% of them were females, and 64.5% of them were married. They worked a mean number of 16.1 shifts in a month (SD = 3.2), including 4.2 night shifts (SD = 1.5) in which they worked 42.3 actual hours per week (SD = 6.1) (Table 1).

**TABLE 1:** Demographic and work‐related characteristics of the participants (*N* = 155).

Characteristic	Mean (SD) or *n* (%)
Continuous variables	
Age, years	32.5 (5.1)
Nursing experience, years	10.2 (3.4)
Scheduled working hours/week	40.8 (5.2)
Actual working hours/week	42.3 (6.1)
Shifts worked/month	16.1 (3.2)
Overtime hours/month	8.5 (2.1)
Night shifts/month	4.2 (1.5)
Categorical variables	
Education	
Diploma	25 (16.1)
Bachelor’s degree	115 (74.2)
Master’s degree	15 (9.7)
Gender	
Male	60 (38.7)
Female	95 (61.3)
Marital status	
Married	100 (64.5)
Single	40 (25.8)
Divorced	10 (6.5)
Widowed	5 (3.2)
Department	
Emergency	40 (25.8)
Intensive care unit	35 (22.6)
General ward	60 (38.7)
Other	20 (12.9)

### 3.2. Descriptive Statistics

The mean overall RCFNRS score was 49.9 (SD = 10.5). Among the four subscales, Recognition and Rewards had the highest mean score (*M* = 18.5, SD = 4.2), followed by Professional Nursing Role (*M* = 15.3, SD = 3.7), Mentoring (*M* = 12.9, SD = 3.1), and Work Adaptability (*M* = 3.2, SD = 1.1) (Table 2).

**TABLE 2:** Descriptive statistics for the Revised Casey–Fink Nurse Retention Survey (RCFNRS) and its subscales (*N* = 155).

Subscale	Mean (SD)
Recognition and rewards	18.5 (4.2)
Professional nursing role	15.3 (3.7)
Mentoring	12.9 (3.1)
Work adaptability	3.2 (1.1)
Overall RCFNRS score	49.9 (10.5)

### 3.3. Correlation Analysis

Pearson correlation analysis revealed statistically significant positive correlations between scheduling flexibility and nurse retention. Specifically, the overall scheduling flexibility score had a strong positive correlation with overall nurse retention (*r* = 0.79, *p* < 0.01). Out of all the individual scheduling practices, the frequency of changing shifts on short notice had the strongest positive correlation with Professional Nursing Role subscale (*r* = 0.79, *p* < 0.01). On the other hand, the possibility of switching shifts on short notice was moderately correlated with Mentoring (*r* = 0.55, *p* < 0.01) (Table 3).

**TABLE 3:** Pearson correlation coefficients between scheduling flexibility variables and nurse retention dimensions.

Scheduling flexibility variables	Recognition and rewards	Professional nursing role	Mentoring	Work adaptability	Overall retention
Possibility to switching shifts on short notice	0.32^∗^	*r* = 0.11^∗^	0.55^∗^	0.18^∗^	0.45^∗∗^
Frequency of changing shifts on short notice	0.49^∗∗^	0.79^∗∗^	0.53^∗∗^	0.23^∗^	0.68^∗∗^
Desire to change current working shift	0.55^∗∗^	0.76^∗∗^	0.31^∗^	0.39^∗∗^	0.44^∗∗^
Influence on planning own shifts	0.45^∗∗^	0.42	0.12	0.42^∗∗^	0.61^∗∗^
Overall scheduling flexibility score	0.68^∗∗^	0.49^∗∗^	0.44^∗∗^	0.61^∗∗^	0.79^∗∗^

*Note:* Pearson correlation coefficients are reported.

^∗^ *p* < 0.05.

^∗∗^ *p* < 0.01.

### 3.4. Regression Analysis

Multiple linear regression analysis showed that six variables significantly predict nurse retention (Table 4). The strongest negative predictor was the number of shifts worked per month (*β* = −0.29, *p* < 0.001), followed by the scheduled working hours per week (*β* = −0.27, *p* < 0.001). Older age (*β* = 0.28, *p* < 0.001) and increased nursing experience (*β* = 0.24, *p* < 0.001) emerged as significant positive predictors for nurse retention. Increasing number of night shifts (*β* = −0.18, *p* = 0.027) and higher actual working hours per week (*β* = −0.16, *p* = 0.015) also predicted lower nurse retention. Overall, the final multiple regression model accounted for 67% of variance in nurse retention (*R* ^2^ = 0.67, adjusted *R* ^2^ = 0.66; *F* = 61.54, *p* < 0.001).

**TABLE 4:** Multiple linear regression predicting nurse retention (*N* = 155).

Predictor	*B*	SE	Standardized *β*	*t*	*p*	VIF
Number of shifts worked/month	−0.20	0.05	−0.29	−4.01	< 0.001	1.30
Age (years)	0.23	0.06	0.28	4.12	< 0.001	1.33
Scheduled working hours/week	−0.18	0.05	−0.27	−3.98	< 0.001	1.18
Nursing experience (years)	0.15	0.04	0.24	3.67	< 0.001	1.25
Night shifts/month	−0.32	0.14	−0.18	−2.23	0.027	1.08
Actual working hours/week	−0.09	0.04	−0.16	−2.45	0.015	1.14

**Model statistics**	***R***	**R** ^2^	**Adjusted** **R** ^2^	***F***		**p**

Final stepwise model	0.82	0.67	0.66	61.54		< 0.001

*Note:* Dependent variable: Overall nurse retention score.

## 4. Discussion

This study found that Jordanian nurses reported low levels of scheduling flexibility and nurse retention, highlighting important workforce challenges within public hospitals. The low mean scheduling flexibility score (*M* = 1.52) suggests that nurses have limited autonomy over their work schedules, particularly regarding shift exchanges and participation in shift planning. This finding is consistent with previous research demonstrating that limited scheduling autonomy reduces job satisfaction and organizational commitment [15]. In the Jordanian context, persistent nursing shortages, increasing patient demand, and staffing constraints likely limit managers’ ability to implement flexible scheduling practices, thereby restricting nurses’ perceived control over their work schedules.

Similarly, the low retention score (*M* = 49.9) indicates deficiencies in key retention domains, including recognition, professional support, mentoring, and work adaptability. These findings are consistent with previous Jordanian studies reporting that inadequate managerial support, limited career development opportunities, and insufficient recognition contribute to lower job satisfaction and increased turnover intentions [13, 16]. These challenges are particularly relevant in resource‐constrained healthcare settings, where heavy workloads often reduce opportunities for professional development and employee recognition.

The present study also demonstrated a strong positive relationship between scheduling flexibility and nurse retention, indicating that greater scheduling flexibility is associated with improved recognition, professional role, mentoring, work adaptability, and overall retention. These findings support previous international studies showing that flexible scheduling enhances work‐life balance, reduces occupational stress and burnout, and strengthens organizational commitment [17, 18]. Flexible scheduling may, therefore, represent an important organizational strategy for improving nurse retention in Jordanian hospitals.

Older age and greater nursing experience were significant positive predictors of nurse retention, whereas longer scheduled working hours, greater actual working hours, more monthly shifts, and more frequent night shifts were significant negative predictors. These findings agree with previous studies demonstrating that experienced nurses are generally more committed to their organizations, while excessive workload contributes to fatigue, burnout, poor work–life balance, and increased turnover intentions [16, 19]. The findings are also supported by international evidence. Studies from Indonesia reported that effective scheduling management improves nurses’ job satisfaction and workforce stability [20], while research from the United States has shown that unstable schedules increase turnover intentions and that adaptive scheduling approaches improve both workforce outcomes and quality of care [21, 22]. Collectively, these studies reinforce the importance of organizational scheduling practices in supporting nurse retention across different healthcare systems.

### 4.1. Recommendations

Based on these findings, healthcare organizations should implement evidence‐based scheduling policies that increase nurses’ participation in shift planning and improve scheduling flexibility while ensuring safe staffing levels. Hospital administrators should strengthen managerial support through recognition programs, structured mentorship, and professional development opportunities, particularly for early‐career nurses. Workforce planning strategies should aim to reduce excessive working hours, frequent night shifts, and workload burden to minimize burnout and improve retention. Future research should employ longitudinal and multicenter designs to examine causal relationships between scheduling practices and nurse retention and to evaluate the effectiveness of flexible scheduling interventions across different healthcare settings.

### 4.2. Limitations

Limitations include the reliance on self‐reported data, which may introduce response bias, and the cross‐sectional design that restricts the ability to infer causality between scheduling flexibility and retention factors. Additionally, the study’s focus on public hospitals may not fully reflect the experiences of nurses in private or specialized healthcare settings. Future longitudinal studies in varied healthcare environments are suggested to validate and expand these findings.

## 5. Conclusion

The study highlighted the significant relationship between nurse managers’ scheduling flexibility and nurse retention in Jordanian public hospitals. It identifies low perceived scheduling flexibility and job retention factors, with sociodemographic and work‐related predictors influencing retention. The findings suggest that improving scheduling practices, managerial support, and work–life balance is essential for enhancing nurse job satisfaction and reducing turnover. This, in turn, can lead to better patient care. Future research is recommended to further investigate these relationships in various healthcare settings and through longitudinal studies for stronger evidence to inform policy and practice improvements.

## Author Contributions

Razan Odaibat: conceptualization. Ghada Abu Shosha, Islam Oweidat, Mutaz I. Othman, Hana A. Alsaeed, Khalid Al‐Mugheed, and Sally Mohammed Farghaly: research design, data collection, analysis, literature search, and manuscript preparation. All authors have accepted responsibility for the entire content of this manuscript and approved its submission.

## Funding

The research was funded by Princess Nourah bint Abdulrahman University Researchers Supporting Project number (PNURSP2026R444), Princess Nourah bint Abdulrahman University, Riyadh, Saudi Arabia.

## Consent

The authors have nothing to report.

## Conflicts of Interest

The authors declare no conflicts of interest.

## Data Availability

All data generated or analyzed during this study are included in this published article.
